# Characterization of Novel Composite Materials with Radiation Shielding Properties for Electronic Encapsulation

**DOI:** 10.3390/ma18245564

**Published:** 2025-12-11

**Authors:** Carla Ortiz Sánchez, Juan José Medina Del Barrio, Gonzalo Fernández Romero, Ángel Yedra Martínez, Paula Ruiz Losada, Luis Alejandro Arriaga Arellano

**Affiliations:** 1 Centro Tecnológico de Componentes—CTC, Scientific and Technological Park of Cantabria (PCTCAN), 39011 Santander, Spain; ayedra@centrotecnologicoctc.com (Á.Y.M.); pruiz@centrotecnologicoctc.com (P.R.L.); 2 ALTER Technology TÜV Nord SAU, C. Tomás Alva Edison, 4, 41092 Sevilla, Spain; jj.medina@altertechnology.com (J.J.M.D.B.); gonzalo.fernandez@altertechnology.com (G.F.R.)

**Keywords:** space radiation, epoxy resin, microelectronic devices, composites, encapsulation

## Abstract

It is well known that the space radiation environment, which has contributions from the trapped particles within the Van Allen belts, solar energetic particles (SEPs) and galactic cosmic rays (GCRs), directly influences space systems. These systems rely on complex and fragile electronic devices, whose performance can be degraded because of the action of the radiation and its related phenomena: single-event effects (SEEs), displacement damages (DDs) and total ionizing dose (TID). This could cause failures to arise through various mechanisms, ranging from parametric drift failures, such as leakage current and threshold voltage, among others, to destructive effects, like single-event burnout (SEB) or single-event latch-up (SEL). These failures in electronics affect the system’s reliability and its performance, which could compromise the mission’s success. Considering this, the main objective of the SRPROTEC project is to develop and validate new composite materials with better shielding performance against space radiation to increase the radiation tolerance of microelectronic devices encapsulated with these materials. For this purpose, three composites will be synthesized using a liquid epoxy resin filled with silica as a matrix mixed in different proportions, with a high-Z filler. The presence of low-Z elements from the high hydrogen content in the polymer and the presence of high-Z fillers are expected to produce a material with good radiation shielding properties. The developed materials will be exhaustively characterized, subjecting the three composites and control samples to rheological outgassing; gamma radiation shielding; and thermal, electrical, thermomechanical and moisture absorption, among other tests. Finally, the composite with the best performance will be selected and subjected to degradation tests (thermal cycling in vacuum, thermal cycling, thermal shock and relative humidity tests) to determine its suitability for space packaging applications.

## 1. Introduction

The radiation environment in space is complex in nature and consists mainly of the following: (i) GCRs, galactic cosmic rays, or high-energy charged particles, such as protons, helium nuclei, electrons and nuclei of heavy atoms; (ii) SEPs or solar energetic particles that consist of electrons and protons; and (iii) Van Allen belt radiation, consisting mainly of electrons and protons [1,2,3]. The radiation from these three contributions poses serious health risks in space exploration and reduces the reliability of electronic components [4].

The effects of space radiation on electronic components used in space missions can be classified into three types: Total Non-Ionizing Dose (TNID), single-event effects (SEEs) and total ionizing dose (TID). TID is due to the accumulation of energy deposited in the material that causes long-term degradation. SEEs consist of specific events where the material is impacted by ions that deposit high amounts of energy in the material, which can lead to the temporary or even permanent malfunctioning of the device. TNID or displacement damage (DD) consists of damage to the lattice structure of solids due to the displacement of atoms when energy is absorbed. These effects can cause malfunctions and/or changes in device settings such as current leakage, increased conduction resistance in transistors, electric arcs, voltage limits, interference, stress, wear and tear and burning of materials, loss of noise immunity, aging and functional and destructive failures [5,6].

Radiation interacts with matter through mechanisms depending on the particle type, energy and material properties. One of the essential characteristics of radiation is its ability to penetrate matter and interact with it. In these interactions, radiation loses some or all of its energy. Among all types of particles in space, this article focuses on photons, uncharged and massless particles [7,8].

**Figure 1:**
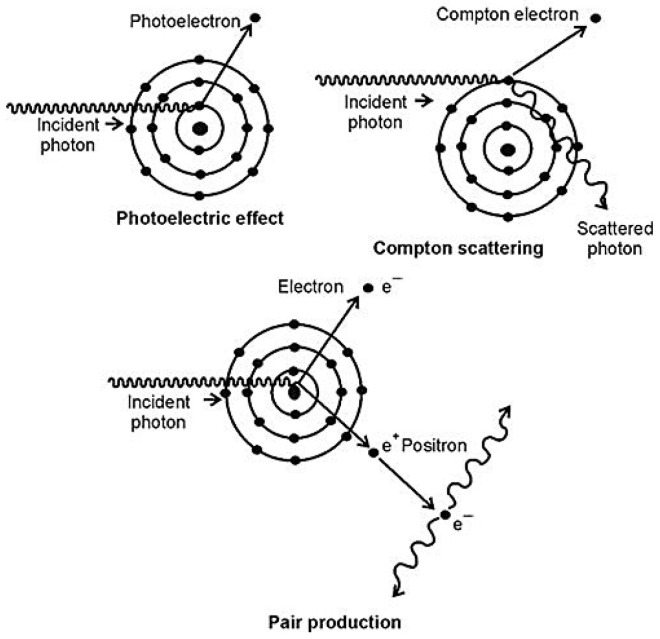
In the photoelectric effect, the photoelectron leaves a vacancy that is filled with an electron from a layer with more energy, emitting ionizing radiation; in Compton scattering, a photon transfers part of its energy and changes its direction; and in pair production, a given photon interferes with the electric field of the nucleus of an atom.

There are three mechanisms by which photons interact with matter and, therefore, lose energy: pair production, the photoelectric effect and the Compton effect (Figure 1) [9].

In the photoelectric interaction [10], when a beam of X-rays or gamma rays penetrates a material, the photon is absorbed and all its energy is passed to an electron, which is expelled as a photoelectron while the photon disappears. The photoelectron leaves a vacancy that is filled with an electron from a layer with more energy, emitting characteristic ionizing radiation. In Compton scattering, a photon transfers part of its energy and changes its direction, while in pair production, a given photon interferes with the electric field of the nucleus of an atom, and the photon vanishes completely while an electron and a positron are created. The attenuation law states that, if a monoenergetic beam of photons (N_0_) falls perpendicularly on a material, there will be a decrease in the number of photons (N) that will depend on the type of material, the thickness (x) and the energy of the incident photons (μ):

(1) $$N=N_{0}e^{-\mu x}$$

**Figure 2:**
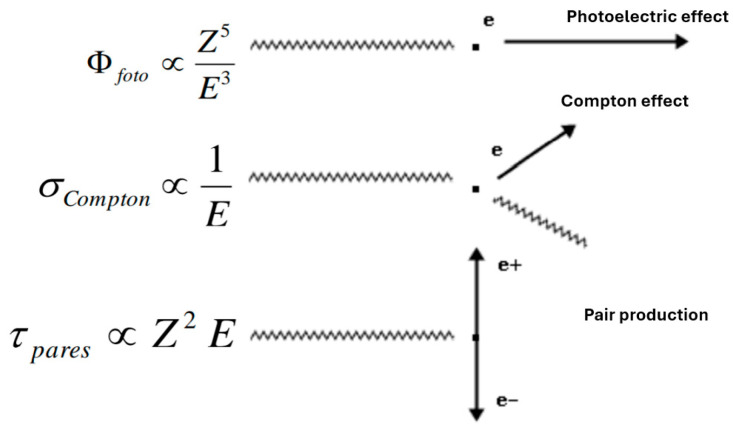
At low energies (X-rays), photoelectric scattering predominates; at medium energies (around 1 MeV), Compton scattering predominates; at higher energies, pair production predominates.

The occurrence of one type of interaction or another will be determined by the energy of the incident photon and the atomic number (Z) of the target material (Figure 2). In general, it can be concluded that, for the attenuation of photon radiation, materials with high Z are more effective as shielding [11].

Shielding is what protects a system or an individual from radiation. The two main types of shielding are active and passive [12,13,14,15,16]:

- Active shielding can be electrostatic, magnetic or plasma.
- Passive shielding aims to create a physical barrier and is currently the most practical approach. The main mechanisms are the absorption and scattering of radiation.

In the microelectronic industry, plastic-encapsulated microcircuits (PEMs) are widely used. These encapsulated circuits [17] have been gaining increasing acceptance due to the advantages offered by their size, weight, cost, availability, performance, technology and design.

Commonly, polymer encapsulants (epoxy, silicone, polyurethane and phenolic resins) with inorganic fillers (silica, alumina, metals) are used to protect components, although currently, the space industry is investing in the search for advanced materials that can function as encapsulants and adhesives to protect microelectronics against space radiation.

Thermosetting and thermoplastic polymers such as polyethylene (PE), polystyrene, epoxy resins, polyethylene terephthalate (PET) and polyamides (PAs), and resins such as polyether, ether ketone, polyimide, polypropylene, etc., have been studied to determine their space radiation attenuation capabilities due to their high hydrogen content, as well as the additives that could be added to improve their capabilities and reduce their degradation. As fillers, the latest research points to the use of certain nano- or micro-additives [18,19], to attenuate or absorb high-energy radiation thanks to the high-volume fraction of particle boundaries, which serve as defect sinks for those produced by ion and proton radiation. When a polymer is reinforced, the probability of an incident photon interacting with the compound increases due to the uniform distribution of the reinforcement over a larger surface area. Furthermore, it may improve the performance of the polymer itself and might allow microelectronics to have a longer lifespan. The goal is to create a shield combining materials with a high and low atomic number (Z) to protect microelectronics from all types of space radiation.

Additives such as aluminum (Al), alumina (Al_2_O_3_), tungsten (W), titanium oxide (TiO_2_), barium oxide (BaO), gadolinium (Gd), graphite, lithium fluoride (LiF), bismuth oxide (Bi_2_O_3_), manganese oxide (MnO), carbon/graphite fibers, hollow glass microspheres or nanoclays can also serve as radiation-shielding fillers. Low-Z materials are more effective at inducing inelastic scattering of electrons and protons, while high-Z materials are more effective at inducing elastic scattering, reducing electron penetration and attenuating photons [20,21,22].

Many of the current studies focus on multilayer structures. For example, Shoorian et al. studied the effect of using a graded multilayer structure based on polyethylene and additives with different atomic numbers to reduce the radiation dose to both the spacecraft crew and electronic components by 110% [23]. Daneshvar et al. developed different multilayer shields for electron and proton space environments [24]. Gohel and Makwana analyzed the efficiency of combining an aluminum slab with layers of lithium hydride, polyethylene, liquid methane, etc., in increasing the shielding effectiveness for SEP and GCR by up to almost 60% [25].

This study, on the contrary, focuses on passive shielding by incorporating high-Z fillers into a polymer matrix with low-Z fillers to develop composites with improved radiation performance suitable for electronic encapsulation. We consider the limitations of the final application in terms of electrical and thermal conductivity and radiation shielding, among others, and seek a solution that is easy to manufacture and apply. In addition, even though the material is designed to shield various types of radiation, the analysis focuses specifically on gamma radiation shielding performance.

## 2. Materials and Methods

The materials used were a commercial, solvent-free, low-coefficient-of-thermal-expansion (CTE), highly thixotropic epoxy resin filled with silicon oxide and a high-Z metal oxide as the filler for its radiation shielding properties, its low electrical conductivity and low CTE, achieving good compatibility with the polymer matrix and microelectronics. High-Z metal oxide is expected to improve the radiation shielding of the epoxy resin against secondary radiation.

**Figure 3:**
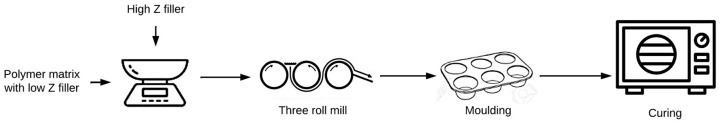
Composite manufacturing process. Firstly, the resin and fillers were hand-mixed, followed by dispersion using a three-roll mill. The composites were then poured into the molds and finally the curing program was carried out.

To prepare the composites (Figure 3), the resin was placed in a breaker and then oxide was gradually added under constant manual stirring in different proportions for each formulation (5 wt.%, 10 wt.% and 15 wt.%). This prevents the formation of particle clusters, which are more difficult to disperse later. Once the mixture was obtained, it was run through two cycles of the three-roll mill. In the three-roll mill, the shear stress created can produce an even dispersion of fillers, which was desired for this application.

Once the mixture was well dispersed, the molds were then filled and cured at 150 °C for 5 min, as indicated in the technical data sheet.

A viscometer (Premium R, Fungilab, Sant Feliu de Llobregat, Spain) at 5RPM was used for viscosity measurements at room temperature for 120 s. As pristine resin is a thixotropic material, the results obtained are comparative.

Following ECSS-Q-ST-70-04C [26], using an inverted metallographic microscope (Olympus GX71, MAB Industrial, S.L.U., Barcelona, Spain), a binocular microscope (Leica Stereozoom S9i, Leica Microsystems, Wetzlar, Germany) and an metallographic microscope (Olympus New VANOX AHMT-513NE, Tokyo, Japan), external visual inspections (EVIs) were carried out to detect surface defects on the samples after the manufacturing process and the degradation tests.

Density measurements were carried out at room temperature using a density balance (GRAM CORE FD, Mateco, S.L., Pontejos, Spain) designed to measure the density of liquids and solids. From these results, the porosity values of the samples were obtained.

The three-point bending tests were carried out on the electromechanical universal testing machine (INSTRON 3369, INSTRON, Cerdanyola del Valles, Spain), taking the UNE-EN ISO 178 standard [27] as a reference for the determination of the flexural properties of plastics at room temperature.

Taking the UNE-EN ISO 62 [28] as a reference, the determination of the absorbed water content after immersion in water at 23 °C was carried out.

Outgassing property characterization was carried out at INTA (Instituto Nacional de Técnica Aeroespacial) in a thermal vacuum outgassing facility accredited by ESA (European Space Agency). Determination of outgassing parameters such as total mass loss (TML), recovered mass loss (RML) and collected volatile condensable material (CVCM) was carried out following ECSS-Q-ST-70-02C [29] in a thermal vacuum equipment model Leybold-ESAVAC under the following conditions: (i) sample temperature (125 °C), (ii) condensable collector temperature (25 °C), (iii) vacuum (1 × 10^−6^–1 × 10^−7^ mbar) and (iv) conditioning of the samples to room temperature before and after the test (22 ± 3 °C, 55 ± 10% RH, 24 h).

Thermal conductivity tests were carried out using a thermal conductivity analyzer (Mathis TCI, Bonsai Advanced Technologies, S.L., Alcobendas, Spain) at room temperature with deionized water as the contact agent.

Thermomechanical measurements to obtain the Coefficient of Thermal Expansion (CTE) and the glass transition temperature (Tg) were performed using a Thermomechanical Analyzer (TMA) (Discovery 450, TA Instruments, New Castle, DE, USA) following the standard ISO-11359-2 [30]. Samples were baked at 105 °C for 2 h before each measurement and then allowed to cool to room temperature under ambient conditions. TMA results were obtained for the first heating run under the following conditions: (i) temperature range (10 to 200 °C), (ii) atmosphere (50 mL/min, N_2(g)_) and (iii) applied force (0.05 N).

Radiation shielding measurements were focused on the characterization of secondary radiation shielding, specifically of gamma radiation. Hence, radiation shielding measurements were carried out at the CNA (Centro Nacional de Aceleradores) facility, using a ^60^Co irradiation source model Gammabeam™ X200 from Best Theratronics (Ottawa, ON, Canada) which emitted gamma rays with characteristic energies of 1.17 Mev and 1.33 MeV, and had an activity of 81,406 TBq at the time of the measurements. A specific system was set up to measure the radiation dose using an ionization chamber from Farmer (PTW, Freiburg, Germany) (model TM30013) with a sensitive volume of 0.6 mm^3^ and an electrometer from PTW (model T10004).

**Figure 4:**
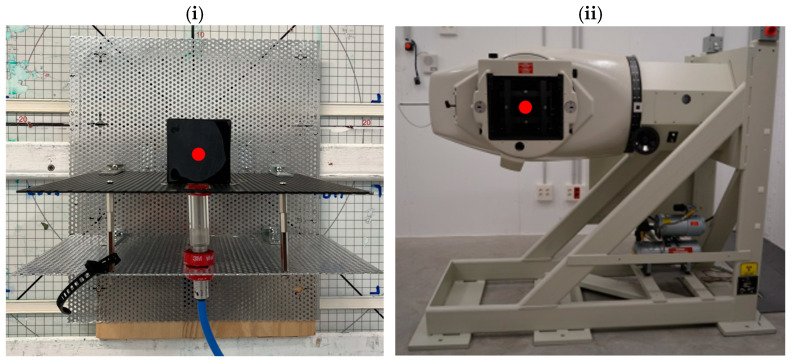
Radiation shielding test set up. Composite sample placed in front of the ionization chamber (**i**) and 60Co irradiator placed in front of the sample (**ii**). The red circles indicate the approximate locations of beam incidence on the sample (**i**) and emission from the source (**ii**).

**Figure 5:**
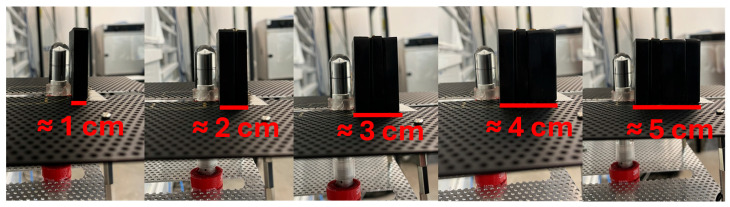
Overlapped samples of composite material in front of the ionization chamber. From one sample up to five samples, from left to right.

Five samples with approximately the same area and thickness were tested by placing them perpendicular to the ^60^Co irradiation source and in front of the ionization chamber (Figure 4). Measurements of the accumulated charge by the ionization chamber, displayed by the electrometer that was connected to it, were carried out in six iterations, starting by measuring without any sample, then measuring one sample, then two samples overlapped and so on until the accumulated charge was measured for five samples (Figure 5) in order to observe the influence of high-Z filler addition on attenuation capacity.

In each one of the iterations, three measurements of the charge accumulated for one minute were registered and the average was calculated. Then, using the N_ki_ conversion factor of the ionization chamber and applying correction factors for pressure and temperature, the accumulated charge registered for each one of the six iterations was converted to the accumulated dose, as shown in the following equation (Equation (2)):

(2) D=Q·Nki·Pc·Tc·100

where D [rad] and Q [C] are the accumulated gamma radiation dose and charge for each iteration, Nki [Gy/C] is the correction factor of the ionization chamber, and Pc and Tc are dimensionless correction factors for pressure and temperature, respectively.

Finally, under the approximations of the radiation beam to be collimated and monoenergetic, Beer–Lambert Law was used to calculate the linear attenuation coefficient for gamma radiation of each material, μ [cm^−1^]. To do so, the ln (I_o_/I) was plotted vs. the thickness of each iteration [cm], where I is the accumulated dose of gamma radiation after penetrating the materials and I_o_ is the accumulated dose registered without any material placed in front of the ionization chamber. This way, μ was calculated as the slope of the plot.

A first set of degradation tests was carried out subjecting the samples to thermal vacuum cycling (TVAC) and temperature cycling (TC) following [26]. In TVAC the samples were tested in the following conditions: (i) temperature range (−65 °C and 150 °C), (ii) nº of cycles (10), (iii) pressure (<1 × 10^−5^ mbar), (iv) residence time (5 min) and (v) temperature rate (1.5 °C/min approximately). In TC the samples were tested in the following conditions: (i) temperature range (−65 °C and 150 °C), (ii) nº of cycles (90), (iii) pressure (atmospheric pressure in N_2(g)_ atmosphere), (iv) residence time (5 min) and (v) temperature rate (10 °C/min). For these measurements, Cryogenic Chamber model 34980A from Sun Electronic Systems and Temperature Measurement System model 34980A from Agilent Technologies were used.

A second set of degradation tests was carried out subjecting the samples to thermal shock (TS) and exposure to relative humidity (RH). The TS was performed following the MIL-STD 202-107 standard [31], using a temperature cycling chamber (air-to-air) model TSE-12 from ESPEC, applying the following conditions: (i) temperature range (−65 to 150 °C), (ii) nº of cycles (100) and (iii) residence time (1 h). The RH exposure was carried out following the MIL-STD 202-103 standard [32], using temperature and humidity chamber model SH-262 from ESPEC and the following conditions: (i) relative humidity (85%) and (ii) temperature (85 °C).

## 3. Results and Discussion

### 3.1. Viscosity Results

Viscosity is the resistance of materials to flow caused by internal friction. In other words, the faster a liquid flows, the lower its viscosity is and vice versa. It is considered an important quantity for the quality control of different substances. Newtonian fluids obey Newton’s law of viscosity and exhibit a linear relationship between angular strain rate and shear stress. Their viscosity remains constant irrespective of the rate of constant stress. In contrast, non-Newtonian fluids do not follow Newton’s viscosity law, and their viscosity decreases or increases depending on the type of fluid under shear. Adding solid particles [33] to a polymer in liquid state changes its viscoelastic behavior, its viscosity and the elasticity of the mixture. These particles act as impeding agents that alter the flow lines of the continuous phase, restricting the mobility of the chains. At high filler levels, the particles are tightly packed, causing particle–particle interactions to predominate over matrix–particle interactions. Constant friction between particles results in energy loss and a higher viscosity mixture is obtained. Also, the viscosity of the mixture increases as the aspect ratio increases for a given filler content.

**Figure 6:**
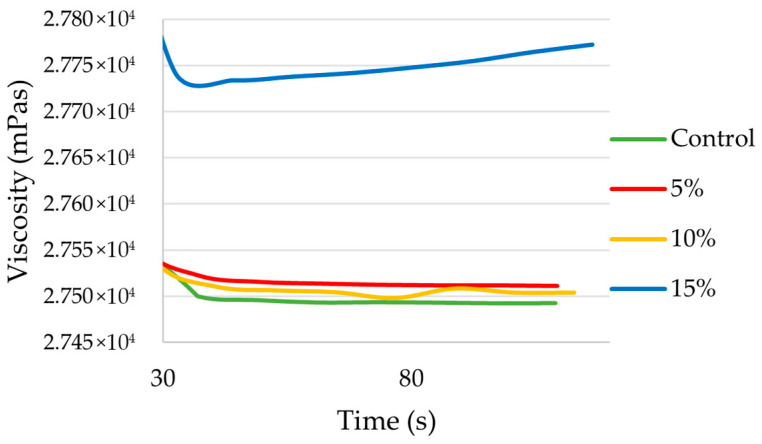
Viscosity variation results (mPas) for control samples (0 wt.% of fillers), 5 wt.%, 10 wt.% and 15 wt.% measured over 120 s.

The results obtained in the viscosity tests show that, although no significant variations in viscosity values have been observed in the set of samples, the 15 wt.% formulation has a slightly higher viscosity than the rest (Figure 6). Although this variation is not very significant numerically, it can affect the actual application of the resin. Although not observed experimentally, a certain change in the performance of the resin is observed visually, with higher filler concentrations showing greater resistance to flow. In addition, the pristine resin initially exhibits thixotropic behavior, and at high concentrations, this begins to change.

### 3.2. Initial Visual Inspections

Some of the main defects found in the specimens prior to any testing were the presence of voids and blistering. The appearance of voids may have been due to a blistering effect where the air in the pore escaped from the material, leaving an unfilled space. The main causes of blistering are as follows [34,35]:

- Solvent or water entrapment.
- Surface contamination.
- Thermal effects due to expansion and contraction.

**Figure 7:**
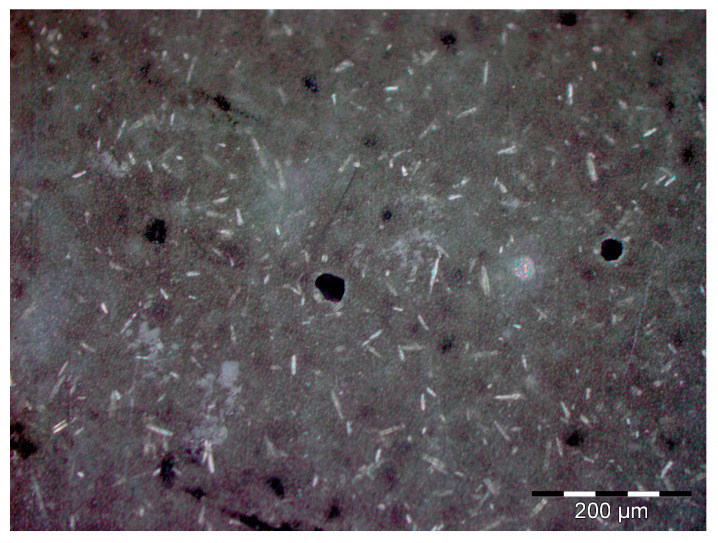

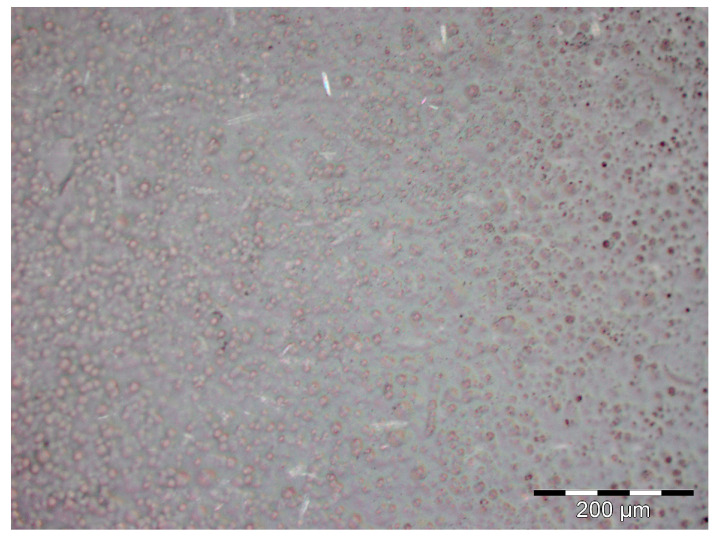
Principal defects observed in the samples before any test. Pores and a rough surface can be observed in the samples.

The main defects observed in the visual analysis of the specimens before being subjected to any type of testing were those inherent to the manufacturing method used (Figure 7). It was observed on the upper face of the specimen (the one that had not been in contact with the mold) that the surface was rough and due to the dispersion method used and the high-temperature curing, voids or pores were produced on the surfaces of the material.

### 3.3. Density, Porosity and Heaviness Results

Once the real density of the different materials was obtained experimentally, the theoretical density of the composites was determined (Table 1) [36]:

(3) $$\rho_{c}=\rho_{m}\left(1-\varphi \right)+\rho_{f}\varphi$$

where ρ_c_ is the theoretical density of the composite (g/cm^3^), ρ_m_ is the real density of the matrix (g/cm^3^), ρ_f_ is the theoretical density of the filler (g/cm^3^) and φ is the volume fraction of the filler. Then, the porosity is determined:

(4) $$p=\frac{\rho_{c}-\rho_{e}}{\rho_{c}}\cdot 100\%$$

where p is the porosity of the material (%), ρ_c_ is the theoretical density (g/cm^3^) and ρ_e_ is the experimental density (g/cm^3^).

Heaviness [37] is another essential property for the application of any shielding material. Taking the aluminum AA6061 as reference material, which has a density of 2.7 g/cm^3^, the percentage of heaviness of the polymer composites was obtained using Equation (5):

(5) $$Heaviness\;\left(\%\right)=\frac{\rho_{material}}{\rho_{aluminum}}\cdot 100$$

**Figure 8:**
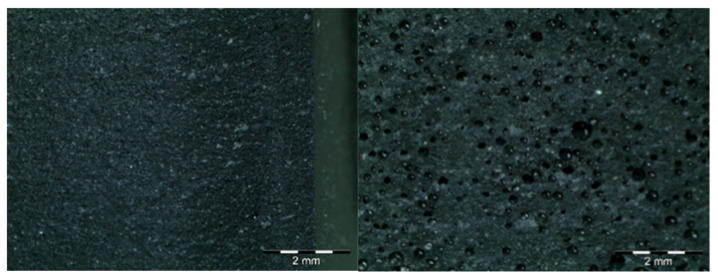
Pore structure observed in the different samples. On the left is the control sample; on the right is the sample with 10 wt.% of metal oxide. It can be observed that when the additive is introduced, the porous structure is more noticeable.

As can be seen in Table 2 and in Figure 8, there is an increase in porosity compared to the control sample. The process of particle incorporation, as well as the presence of moisture in the particles and the formation of agglomerates, introduces air into the matrix and generates the porous structure. In the manufacturing process, primary bubbles can be created in the mixing process and secondary bubbles in the curing process. The porosity of the composites produces a variation in density when compared to the control sample (Table 1) and compared to what should be the theoretical density of the test probes at a porosity of 0%.

### 3.4. Mechanical Strength Results: Maximum Stress

The addition of fillers can improve many properties, such as mechanical strength, thermal and electrical conductivity and thermal stability, but it can also decrease certain properties when a critical proportion of solid fillers is added, such as impact strength or flexural strength. Material cohesion is reduced with the addition of high filler contents, but stiffness increases, resulting in higher storage and tensile and flexural moduli.

As Table 3 shows, the samples with higher filler content have higher porosity than the control sample, and their mechanical properties are worse. This is consistent with the general theory that the higher the porosity, the worse the mechanical properties [38], as these pores act as stress points.

### 3.5. Water Absorption Results

The absorption of moisture in polymers can cause a significant increase in weight, resulting in a loss of structural integrity in critical aerospace materials [39,40,41]. This change induced by moisture not only affects weight but also has a significant impact on the mechanical properties of polymers, including their tensile strength and elasticity. Moisture’s impact is mainly manifested through a reduction in the material’s strength. Absorbed moisture reduces the glass transition temperature and the associated degradation of high-temperature properties, even with minimal moisture exposure. This decrease in Tg is due to the breaking of strong hydrogen bonds in the cured network, and their replacement by weaker hydrogen bonds related to water. Additionally, moisture absorption leads to volumetric expansion. Even materials traditionally considered dimensionally stable can exhibit unexpected behavior in high-precision assemblies.

Results in Table 4 show that the higher the concentration of fillers, the higher the water absorption. Also, these results are related to the porosity values. It is well known that the curing process of epoxy resins is an exothermic reaction, and in that exothermic reaction, the water contained in the resin evaporates and produces bubbles during curing, which then results in pores [38].

### 3.6. Outgassing Results

Outgassing [42] is the property of non-metallic materials that, under high-temperature and/or low-pressure conditions, release vapors by sublimation that can condense in adjacent systems, rendering them inoperable. The degree of outgassing [43] depends on the material and its characteristics, along with the temperature and the amount of time the material remains in vacuum. Total mass loss (TML) is the mass loss of a sample expressed as a percentage compared to the initial mass. Recovered mass loss (RML) is the mass loss of a sample expressed as a percentage compared to the initial mass after 24 h in a vacuum, which is followed by 24 h of conditioning. Collected volatile condensable material (CVCM), expressed as a percentage, is the contaminant mass on a collector plate above a material sample after 24 h in a vacuum environment.

It is well known that drastic pressure reduction and temperature oscillations can affect the physical stability of polymeric composites and cause outgassing phenomena [44]. The ultra-high vacuum level of common space operating environments can cause sublimation of exposed surface atoms leading to structural weakening and contamination.

As shown in Table 5, all the samples tested are compliant with general limits of acceptance for material selection according to ECSS-Q-ST-70-02C, where RML and TML < 1.00% and CVCM < 0.10%. While CVCM seems to be independent of the filler content, TML and RML decrease with it. This might be linked to the reduction in the volume fraction of the polymeric matrix, making the composite less susceptible to outgassing phenomena.

### 3.7. Thermal Conductivity Results

In microelectronics, a very common problem is heating dissipation. This heat must be dissipated as quickly as possible to keep the operating temperatures at the desired level and thus increase the lifespan. In solid materials, heat can be transported by phonons, that is, the quantity of energy from the vibrations of the atomic lattice, or by charge carriers, such as electrons or holes. In semiconductors and insulators, such as the control and composite materials investigated, the thermal conductivity is dominated by phonons, whereas in conducting materials, it is dominated by electronic contribution.

Most polymers have low thermal conductivity, around 0.1–0.5 W/mK [45]. For the control sample, the thermal conductivity measured was higher (0.9 W/mK), which agrees with the fact that it is not a resin based only on polymeric materials; it is an epoxy resin with a high content of SiO_2_, which has a thermal conductivity of 1.3 W/mK on its own [46]. Hence, the value of the control sample is slightly higher than that in a polymer without additives. The type, proportion, size and shape of the filler have a strong influence on the thermal conductivity of polymeric composites, as well as their spatial arrangement and orientation.

As can be seen from the results obtained in Table 6, there is neither an improvement with respect to the reference thermal conductivity nor a linear increase with respect to the higher filler concentration in the composites. These results might be related to the increase in the viscosity of the composites with the introduction of new fillers, which has led to an increase in porosity and therefore a lower thermal conductivity, as the air contained in the pores acts as a thermal insulator.

### 3.8. TMA

CTE and Tg (glass transition temperature) are critical properties for electronic encapsulation materials. The packaging material protects the internal electronic components, and it is in direct contact with materials such as metals, alloys and semiconductor materials like silicon, among others. CTE mismatches between different materials combined with temperature variations is one of the main issues for reliability in the semiconductor industry [47]. This combination can induce stress in the materials’ interfaces that could lead to delamination issues and is the cause of significant mechanical stress accumulation in the die surface and wire bond fracture [48,49]. The Tg is the representative temperature of the temperature range where the glass transition takes place, which is a reversible change from a hard and brittle state into a rubbery state [50]. The importance of this property relies on the fact that during this temperature range, and once it is exceeded, physical properties of the material like mechanical and dielectric properties, CTE and viscosity, among others, drastically change.

For the reasons explained above, it is necessary to characterize the CTE and Tg values of the composite materials. Table 7 shows the CTE and Tg results of all the samples measured for the four formulations.

As shown in Table 7, CTE 2, corresponding to the rubbery state (T > Tg), is on average 2.63 μm/m°C times greater than the CTE1, corresponding to the glassy state (T < Tg). This increase agrees with typical increases in the CTE of epoxy-filled systems when the composite exceeds Tg [51] and reaches the rubbery state. Also, CTE1, CTE2 and Tg are within the expected values for epoxy-filled systems used for packaging applications [52,53,54].

When comparing the different formulations, it can be observed that CTE 1 of the control sample decreases as the filler proportion increases. A similar trend is observed for CTE 2, except for the 15 wt.% formulation, where the reduction is less pronounced than in the 10 wt.% formulation relative to the control sample. The general trend can be explained by the increase in high-Z fillers (with low CTE) in the volume of the composite, which contributes to the decrease in the overall CTE. The exception might be related to a higher presence of voids in the 15 wt.% formulation, which could influence the CTE [55,56] by increasing it, counteracting the CTE 2 reduction associated with filler addition.

For Tg, the dependency on filler content does not show a clear trend. When compared with the control formulation, Tg for the 5 wt.% formulation remains almost the same; for 10 wt.%, it decreases; and for 15 wt.%, it increases. These variations could be associated with how the fillers interact with the polymeric matrix and how the quantity of fillers affects the crosslinking of them.

### 3.9. Radiation Shielding Test

**Figure 9:**
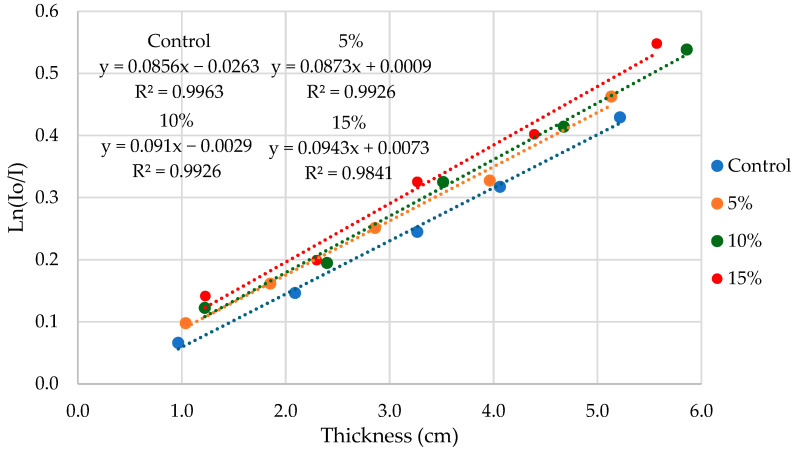
ln (I_o_/I) plotted vs. thickness for each composite formulation. Results suggest that epoxy matrix μ is improved by the addition of high-Z fillers and that μ increases with the addition of these fillers.

The radiation shielding test was focused on characterizing the shielding properties of the synthesized composites and control samples against gamma radiation. As can be seen in Figure 9, the composite formulations with higher slopes were the ones with a higher linear attenuation coefficient, μ [cm^−1^]. The results suggest that the higher the filler concentration, the higher the μ. Hence the gamma radiation shielding improves with the high-Z filler concentration.

**Figure 10:**
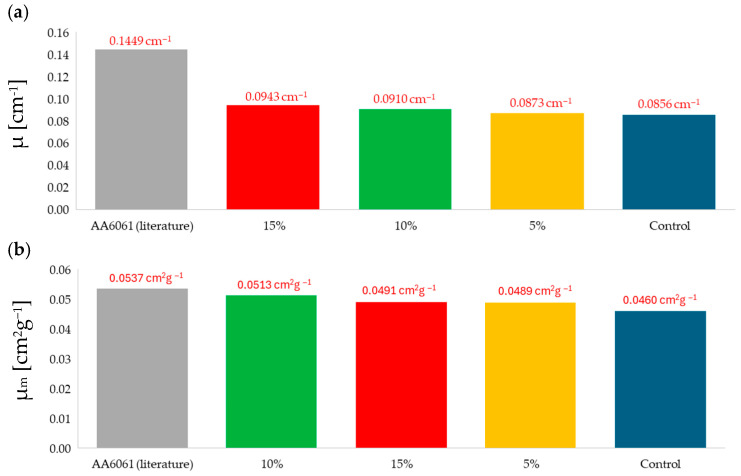
μ (**a**) and μ_m_ (**b**) for the composite formulations and the AA6061 reference [57]. This comparison reveals that the composites do not reach the gamma radiation shielding level of the AA6061 for neither μ nor μm.

In Figure 10a, all the μ are compared, while in Figure 10b, the mass attenuation coefficient, μ_m_ [cm^2^g^−1^], is shown. μ_m_ is obtained by dividing μ by the experimental density of the composites: μ/$\rho_{m}$.

If μ is compared across all the formulations, results suggest that the epoxy matrix μ is improved by the addition of high-Z fillers, and that μ increases with the addition of these fillers. However, if μ_m_ is compared, the 10 wt.% formulation outperforms the 15 wt.% The μ_m_ for the 10 wt.% formulation increases and exceeds that of the 15 wt.% formulation because the 10 wt.% composite is less dense due to its porosity.

Figure 10a,b also show the comparison between the different formulations and the μ and μ_m_ of the AA6061 [57], an alloy widely used for space applications [58]. A comparison with aluminum, the most widely used material in space applications for radiation shielding, allows the developed composites to be assessed against the standard reference material for this specific application. This comparison reveals that the composites do not reach the gamma radiation shielding level of the AA6061 for neither μ nor μ_m_. However, given the lower density of the composites compared to the aluminum’s density, μ_m_ values for the composites are close to the AA6061 ones. If porosity was reduced and density slightly increased, μ_m_ could decrease, but at the same time, the densification of the composite could increase the μ, compensating for the μ_m_ reduction. If the manufacturing process was refined, the developed composites could improve their radiation shielding properties, matching or outperforming the AA6061. 

Table 8 summarizes the improvement in the linear attenuation coefficient and the mass attenuation coefficient of the samples with 5 wt.%, 10 wt.% and 15 wt.% of fillers compared to the control sample without any additive.

A. Final formulation selection

Viscosity and radiation shielding properties (μ_m_ and μ) were considered as some of the most relevant properties to select the final material. Viscosity is a key parameter for encapsulation; if it is excessively high, it can damage the wires of the electronic component, and it can also lead to an incomplete encapsulation of the intricate geometries. Regarding radiation shielding, the improvement in this property was the main objective of this project. The 10 wt.% and 15 wt.% composites were the best candidates for radiation shielding improvement, for which the highest values of μ_m_ and μ were obtained. The 15 wt.% formulation may have looked like a good candidate to be selected, but its viscosity value was too high for the intended dam and fill application. The 10 wt.% formulation showed an acceptable viscosity value, like the control sample’s viscosity, and exhibited the highest value of μ_m_ and the second highest value of μ. These results make the 10 wt.% formulation the optimal balance between the radiation shielding effectiveness and processability properties.

Regarding the porosity, it is worth mentioning that despite the 10 wt.% formulation having the highest value of porosity, if it was decreased, density and μ_m_ would increase and decrease, respectively, but at the same time, μ might increase because of the reduction in air voids in the polymeric matrix. Considering this, the 10 wt.% filler formulation could be improved further than the 5 wt.% formulation. Its higher initial attenuation values (μ) despite its higher porosity suggest that reducing voids and improving densification might lead to an improvement in the performance of the composite, likely outperforming the 5 wt.% in both μ and μ_m_ if the porosity was reduced for this formulation as well.

The maximum flexural strength recorded for all formulations was the lowest in the 10 wt.% formulation. However, this formulation also exhibited the lowest standard deviation, while the control and 5 wt.% formulations showed particularly high variability. It is possible that the maximum bending stress values may have not completely and precisely represented the full set of formulations, reducing the accuracy of direct comparisons between them.

Water absorption for the 10 wt.% formulation increased when compared to the control and 5 wt.% formulations, but it remained lower than the water absorption value of the 15 wt.% formulation. This formulation had a level of water absorption 14.5 times greater than the water absorption of the control formulation, while water absorption of the 10 wt.% formulation was only 5 times greater than the water absorption of the control sample.

Although thermal conductivity for the 10 wt.% formulation was slightly lower than the thermal conductivity for the control sample (0.865 W/mK vs. 0.990 W/mK), it showed better results than the 5 wt.% and 15 wt.% formulations.

Regarding the TMA results, CTE (T < Tg) was like the CTE (T < Tg) of the other formulations, showing good dimensional stability, while CTE (T > Tg) was the lowest among all the formulations, showing the best dimensional stability. Tg was the lowest of all the formulations, but its value was still acceptable for electronic packaging applications [59,60].

As for the other formulations, the 10 wt.% formulation complied with the outgassing limits specified in the ECSS-Q-ST-70-02C standard and exhibited similar surface defects following the manufacturing process.

Finally, based on the characterization results, the composite containing 10 wt.% fillers demonstrated the most favorable balance between radiation shielding effectiveness and material properties for packaging applications. Hence, this formulation was selected as the final composite to be subjected to the degradation test sequences.

B. Degradation tests

(1) TVAC and TC results: TMA, EVI and Flexural Strength

Materials with space applications are subjected to thermal cycles and in many cases to ultra-high-vacuum conditions. These harsh conditions can significantly accelerate the materials’ degradation, including epoxy-based materials [61,62]. Moreover, for the composite developed, due to the differences in the CTE between the additives and the polymer matrix, stresses can repeatedly form under changing temperature conditions caused by the temperature cycling, which can lead to interfacial detachment, microcracking and increased degradability of the material [63,64]. Hence, it is vital to study the composite’s degradation under these harsh conditions. Samples are characterized before and after the degradation tests via visual inspection, thermomechanical analysis (CTE and Tg) and flexural strength measurements (maximum bending stress).

Due to the importance of CTE and Tg in packaging applications, these properties are selected as control properties to be assessed before and after the thermal vacuum test. Table 9 shows TMA results before and after TVAC + TC tests for the samples subjected to this test sequence.

CTE for both temperature ranges, before Tg and after Tg, decreased after the TVAC test, while Tg showed an important increase. The remarkable increase in Tg could be associated with an increase in the crosslinking degree of the polymeric matrix of the composite, caused by the post-curing [65,66,67] that occurred in the TVAC + TC test at high temperatures. CTE decrease could also be associated with the higher crosslinking degree reached during post-curing, which would lead to chain mobility reduction, lowering CTE values. Another factor that could have contributed to the decrease in CTE is the loss of polymeric matrix of the composites during TVAC due to outgassing of volatile components in vacuum conditions, which translates into a composite material with a lower volume fraction of polymeric matrix and a greater volume fraction of ceramic fillers with lower CTE, decreasing the global CTE of the composite.

Figures from Table 10 show visual inspection results, which do not indicate degradation associated with the TVAC + TC test sequence. Although voids or microcracks could be expected due to the outgassing-induced porosity during the TVAC test, such defects were not found in the surfaces of the samples.

Table 11 shows that the maximum bending stress slightly decreases after TVAC + TC. This might be linked to degradation of the composite due to the TVAC + TC test. However, it is most likely that the notable presence of porosity (12.64% for the 10 wt.% formulation) influenced the results as well. If the samples were measured in a volume with a high porosity concentration in the final mechanical measurements, the decrease in the maximum bending stress might have been related to the presence of porosity as well, and not only to TVAC + TC-induced degradation.

Results disclose that the composite formulations with 10 wt.% fillers do not significantly degrade when exposed to TVAC and TC. No degradation was observed on the surface of the samples and the decrease in the maximum bending stress was low and possibly linked to the porosity of the composite associated with the manufacturing. Finally, the observed changes in CTE and Tg were not detrimental to the intended application. The increase in Tg expanded the temperature range within which the material could be safely used without significant changes in its properties, while the reduction in CTE reduced mismatch with other materials used in electronic components.

(2) Thermal Shock (TS) and Relative Humidity (RH) Exposure: TMA, EVI and Flexural Strength

When selecting materials for space applications, it is essential to not only consider the space conditions but also those on Earth during manufacturing and storage. Although humidity is not present in the space environment, its impact on material degradation was assessed due to potential exposure prior to launch. To study degradation behavior, the composite was subjected to a test sequence consisting of TS followed by RH exposure. As was conducted for the TVAC + TC test sequence, to control the possible degradation of the material, TMA, visual inspection and flexural strength measurement characterizations were performed before and after the environmental tests.

As Table 12 shows for TMA characterization of samples subjected to TS + RH, changes in CTE and Tg were registered: CTE 1 slightly increased, Tg increased and CTE 2 decreased. Internal microcracks might be the cause of CTE 1’s slight increase, allowing greater expansion of the composite, while the Tg increase might have been caused by a possible post-curing process that took place during the TS test at temperatures higher than Tg, increasing the crosslinking density of the polymeric matrix [42]. CTE 2’s decrease could be explained using the same reasoning of the Tg increase; if post-curing took place during the TS, segmental mobility was reduced even above Tg, leading to a lower value of CTE 2 in the final TMA measurement.

As shown in Table 13, the visual inspection results shown did not indicate degradation associated with the exposure to TS + RH.

The maximum bending stress shown in Table 14, as in the case of the TVAC + TC test sequence, slightly decreased after the exposure to TS and RH. The reasoning that could explain this decrease is similar to the reasoning that could explain the decrease in the mechanical properties after TVAC + TC. It might have been related to degradation caused by the exposure to the environmental tests, but it also could have been influenced by the high porosity of the 10 wt.% formulation.

These results suggest that the composite formulation with 10 wt.% fillers did not significantly degrade when it was subjected to TS and RH exposure. Similarly to the TVAC + TC test sequence, the maximum bending stress decrease was small (and might have also been related to the porosity of the composite produced during its manufacturing) and no degradation was evidenced on the surface of the samples. Regarding the CTE and Tg variations, as explained in Section 1 TVAC + TC results: TMA, EVI and Flexural Strength, Tg and CTE 2 increases were positive for the intended packaging application. CTE 1’s increase remained the only variation after the degradation tests that might have been considered detrimental for the application due to the increase in the mismatch with other materials of the electronic components, but the increase registered was not high.

## 4. Conclusions

The space radiation environment can cause failures in electronic systems that can affect the space system’s reliability and its performance, which could compromise the mission’s success. Passive shielding solutions based on the interaction of radiation with matter can mitigate the effects of space radiation. In this regard, the SRPROTEC project focused on the development and study of composite materials based on a polymeric matrix with 5 wt.%, 10 wt.% and 15 wt.% of high-Z fillers, to determine if they could be (i) used as packaging materials with enhanced radiation shielding properties for electronic components and (ii) be a potential substitute for aluminum for shielding applications in space due to their lower density.

A screening was performed, assessing relevant physical properties of all the formulations to determine which one performed better as a package material for electronic systems while showing enhanced gamma radiation shielding properties. Certain changes were observed in the rheological properties of the samples in the initial screening, leading to alterations in their porosity, density and water absorption. This, in turn, led to some changes in their thermal properties and outgassing values, which were still acceptable according to the ECSS-Q-ST-70-02C criteria. Finally, in this initial screening, the gamma radiation shielding capability of the composites was studied, confirming that the shielding against gamma radiation was improved by the high-Z filler addition to the epoxy matrix. Based on the screening results, the formulation containing 10 wt.% of fillers was identified as the option that showed the best balance between properties relevant for packaging and gamma radiation shielding capabilities. The variation in viscosity compared to the control sample was negligible; compared to the reference aluminum, the density of the protective material could be reduced from 2.7 g/cm^3^ to 1.77 g/cm^3^ and a linear attenuation coefficient value of 0.0910 cm^−1^ was achieved.

The selected formulation was subjected to two different sequences of degradation tests: (i) TVAC + TC and (ii) TS + RH. Results disclosed that the selected formulation did not significantly degrade when exposed to TVAC and TC, nor to TS or RH exposure. Before any degradation test, the maximum bending stress was 33.53 MPa, and after the TVAC and TC test, it was 31.34 MPa; similarly before the TS and RH test, the maximum bending stress was 31.87 MPa.

Finally, the values of μ_m_ obtained for the composites were close to the μ_m_ of the AA6061 (0.0537 cm^2^g^−1^), with the final formulation (containing 10 wt.% of high-Z fillers) exhibiting the closest match, presenting a μ_m_ of 0.0513 cm^2^g^−1^. If the manufacturing process was enhanced and porosity was decreased, gamma radiation shielding properties of the composites could improve, matching and even outperforming AA6061 values. Additionally, manufacturing improvements and consequent porosity reduction could reduce the risk of failures related to the presence of voids when the material is used for electronic component encapsulation. Future work could also involve a more extensive study of the composite’s shielding properties against primary radiation.

## Figures and Tables

**Table 1:** Density results.

Sample	Density (g/cm^3^)	SD (g/cm^3^)	Theoretical Density (g/cm^3^)
Control	1.860	±0.010	1.87
5%	1.787	±0.040	1.94
10%	1.773	±0.021	2.03
15%	1.920	±0.035	2.12

**Table 2:** Porosity and heaviness results.

Sample	Porosity (%)	SD (%)	Theoretical Heaviness (%)
Control	0.53	±0.53	69.26
5%	7.90	±2.08	71.85
10%	12.64	±1.02	75.18
15%	9.43	±1.63	78.51

**Table 3:** Mechanical property results.

Sample	Maximum Bending Stress (MPa)	SD (MPa)
Control	51.40	±23.81
5%	52.06	±14.38
10%	33.53	±4.37
15%	36.12	±7.43

**Table 4:** Water absorption results.

Sample	Water Absorption (%)	SD (%)
Control	0.02	±0.015
5%	0.08	±0.006
10%	0.10	±0.006
15%	0.29	±0.130

**Table 5:** Outgassing results.

Sample	TML (%)	SD (%)	RML (%)	SD (%)	CVCM (%)	SD (%)
Control	0.412	±0.004	0.295	±0.003	0.003	±0.002
5%	0.349	±0.021	0.205	±0.019	0.003	±0.003
10%	0.323	±0.010	0.171	±0.025	0.005	±0.001
15%	0.277	±0.013	0.170	±0.008	0.003	±0.001

**Table 6:** Thermal conductivity results.

Sample	Thermal Conductivity (W/mK)	SD (W/mK)
Control	0.990	±0.16
5%	0.805	±0.06
10%	0.865	±0.08
15%	0.816	±0.03

**Table 7:** TMA results of the four formulations.

Formulations	Control	5%	10%	15%
CTE 1 (T < Tg) (μm/m°C)	19.27	18.01	17.84	17.53
SD (μm/m°C)	±0.91	±0.90	±1.22	±0.70
Tg (°C)	135.46	135.08	130.91	144.7
SD (°C)	±0.63	±3.44	±4.94	±6.05
CTE 2 (T > Tg) (μm/m°C)	51.6	47.82	44.88	46.5
SD (μm/m°C)	±4.14	±1.39	±1.01	±2.78

**Table 8:** Observed improvement in the linear attenuation coefficient and the mass attenuation coefficient of the samples with different contents of fillers compared to the control sample.

Sample	Improvement in μ (cm^−1^) Compared to the Control Sample (%)	Improvement in μ_m_ (cm^2^g^−1^) Compared to the Control Sample (%)
5 wt.%	1.99	6.15
10 wt.%	6.31	11.52
15 wt.%	10.16	6.72

**Table 9:** TMA results before and after TVAC + TC tests for 10 wt.% samples.

Measurement	Initial Measurement (Before TVAC + TC)	Final Measurement (After TVAC + TC)	Difference Between Final and Initial Measurement
CTE 1 (T < Tg) (μm/m°C)	18.49	16.88	−1.62
SD (μm/m°C)	±0.94	±0.26	-
Tg (°C)	134.74	146.13	11.39
SD (°C)	±4.13	±1.80	-
CTE 2 (T > Tg) (μm/m°C)	45.47	43.35	−2.12
SD (μm/m°C)	±0.91	±1.96	-

**Table 10:** Visual inspection results before and after the TVAC + TC test for a 10 wt.% sample (20× magnification). No damage is visible to the samples.

Visual Inspection	
**Initial Visual Inspection (before 10 TVAC cycles)**	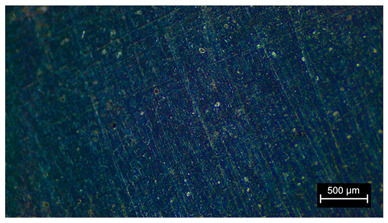
**Final Visual Inspection (after 10 TVAC and 90 TC cycles)**	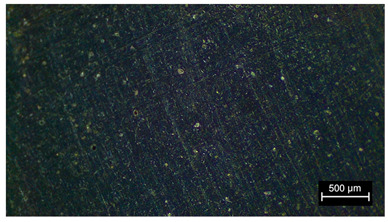

**Table 11:** Maximum bending stress results before and after TVAC + TC for 10 wt.%. samples.

Property	Initial Measurement (Before TVAC)	Final Measurement (After TVAC)	Difference Between Final and Initial Measurement
Maximum Bending Stress (MPa)	33.53	31.34	−2.2
SD (MPa)	±4.37	±9.27	-

**Table 12:** TMA results before and after TS + RH tests for 10 wt.% samples.

Measurement	Initial Measurement (Before TS + RH)	Final Measurement (After TS + RH)	Difference Between Final and Initial Measurement
CTE 1 (T < Tg) (μm/m°C)	17.18	18.04	0.86
SD (μm/m°C)	±1.12	±0.40	-
Tg (°C)	127.08	131.68	4.60
SD (°C)	±1.54	±1.74	-
CTE 2 (T > Tg) (μm/m°C)	44.29	41.07	−3.22
SD (μm/m°C)	±0.71	±3.82	-

**Table 13:** Visual inspection results before and after the TS + RH test for a 10 wt.% sample (20× magnification). No damage is visible to the samples.

Visual Inspection	
**Initial Visual Inspection (before TS + RH)**	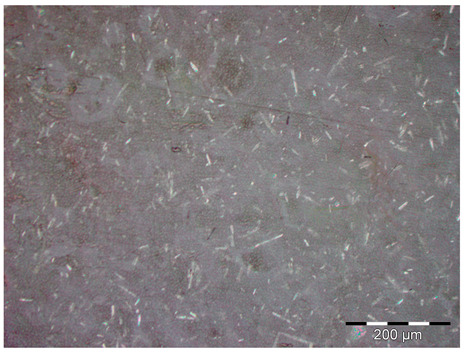
**Final Visual Inspection (after TS + RH)**	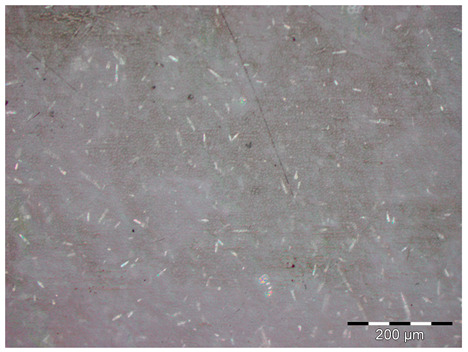

**Table 14:** Maximum bending stress results before and after TS + RH tests for 10 wt.% samples.

Property	Initial Measurement (Before TS + RH)	Final Measurement (After TS + RH)	Difference Between Final and Initial Measurement
Maximum Bending Stress (MPa)	33.53	31.87	−1.7
SD (MPa)	±4.37	±6.00	-

## Data Availability

The original contributions presented in this study are included in the article. Further inquiries can be directed to the corresponding authors.
